# The association of material deprivation component measures with injury hospital separations in British Columbia, Canada

**DOI:** 10.1186/s40621-019-0198-7

**Published:** 2019-06-10

**Authors:** Fahra Rajabali, Alex Zheng, Kate Turcotte, Li Rita Zhang, Diana Kao, Drona Rasali, Megan Oakey, Ian Pike

**Affiliations:** 1 Department of Pediatrics, University of British Columbia, Vancouver, British Columbia Canada; 2 BC Injury Research and Prevention Unit, BC Children’s Hospital Research Institute, Vancouver, British Columbia Canada; 3 BC Centre for Disease Control, Provincial Health Services Authority, Vancouver, British Columbia Canada; 4 Faculty of Kinesiology and Health Studies, University of Regina, Regina, Saskatchewan Canada

**Keywords:** Injury hospital separations, Socio-economic status, Deprivation indices, Income, Education, Employment, Injury priorities

## Abstract

**Background:**

This study examines social disparities across neighbourhood levels of income, education and employment in relation to overall injury hospital separations in the province of British Columbia, Canada. Further, the study examines the relationships of social disparities to a set of three injury prevention priorities in British Columbia, namely, transport (motor vehicle occupant, pedestrian and cyclist), falls among older adults, and youth self-harm. The goal being to better understand area-based injury incidence with a view to precision prevention initiatives, particularly for more vulnerable populations.

**Methods:**

Acute hospital separations from the Discharge Abstract Database were identified for all causes of injury and the three BC injury prevention priorities for the period April 1, 2009 to March 31, 2014, inclusive. An ecological approach was applied where each hospital separation case was attributed with the income, education and employment level according to the injured individual’s area of residence, derived from the 2011 CensusPlus data.

**Results:**

Injury hospital separation data were available for 191 Forward Sortation Areas in BC. Between April 1, 2009 and March 31, 2014, there was a total of 177,861 injury-related hospital separations, averaging 35,572 hospital separations per year and an annual rate of 779 injury hospital separations per 100,000 population. Injury hospital separation rates varied with the measured neighbourhood area socioeconomic status variables. Injury hospital separation rates demonstrated an inverse relationship with neighbourhood levels of income and education. Neighbourhood area socioeconomic status differences were also associated with the injury hospital separation rates for falls among older adults, motor vehicle crashes involving motor vehicle occupants, pedestrians, cyclists and young drivers, and youth self-harm.

**Conclusions:**

The study results show that neighbourhood levels of income, education and employment are associated with the risk of injury hospital separation. In particular, low education levels in FSAs was associated with increased risk of injury hospital separation, mainly for motor vehicle occupants, pedestrians, young drivers, and youth self-harm. The results of this study provide useful information for implementing injury prevention initiatives and interventions in BC to align with the provincial public health system and road safety strategy goals, particularly for identified priorities.

## Background

Injuries are the leading cause of death among British Columbians aged 1 to 44 years (Statistics Canada 2018). Injuries in British Columbia (BC) resulted in 2110 deaths, 37,207 hospital separations, 482,687 emergency room visits, 8911 permanent disabilities, and cost British Columbians $4.1 billion in healthcare related costs in 2013 (Rajabali et al. 2018). Research shows that patterns of injury can be identified on the basis of age, sex, gender, health status, contributing factors, social characteristics and geographic location (Rivara and Mueller 1987). Specific causes of injury have been found to vary with age, and higher incidence rates of injury hospital separations are observed for children, youth and older adults. In BC, injuries from falls and transport incidents have resulted in the highest number of years lived with disability, while unintentional poisoning and injuries from self-harm have resulted in the highest number of years of life lost, with the gross cost per disability-adjusted life years for these causes ranging from $547 million to $922 million (Rajabali et al. 2018). Most injuries are considered to be preventable, as they are the result of predictable and avoidable risk factors and not merely chance occurrences. By understanding the patterns and risk factors of injury, targeted prevention programs can be designed to reduce the burden of injury.

A large proportion of scientific literature focuses on injury as it relates to socioeconomic status (SES) among adolescents and children (Birken et al. 2006, Faelker et al. 2000, Gagne and Hamel 2009, Oliver and Kohen 2010, Pickett et al. 2005, Simpson et al. 2005). A recent study in BC has shown significant disparities in unintentional and intentional injury-related mortality outcomes between the sexes and by SES (Zandy et al. 2019). Some studies have focused on severe injury and trauma, investigating the relationship with SES (Amram et al. 2015, Lawson et al. 2015). Other studies have focused on the relationship between SES and specific mechanisms, such as assault (Bell et al. 2009), self-harm (Eggertson 2013), falls (Jin et al. 2017), playground injuries (Macpherson et al. 2010), road traffic injuries (Morency et al. 2012, Oliver and Kohen 2009) and dog bites (Raghavan et al. 2014). A significant inverse relationship between SES and injury has been demonstrated where people with lower SES groups have higher hospital separation rates, almost 1.3 times higher, compared with people from higher SES groups (Canadian Institute for Health Information (CIHI) 2010).

Similar socio-economic gradients were observed for both males and females, although hospital separation rates were higher for males than females. Furthermore, a SES gradient is evident when considering different causes of injury, including traffic-related injuries, suicide attempts, assaults, unintentional poisonings, as well as injuries occurring at home, on the road, or at work (Birken snd Macarthur 2004, Canadian Institute for Health Information (CIHI) 2010).

In 2017, the British Columbia Injury Prevention Committee (BCIPC) was formed and included health authority, public health and Ministry of Health representatives, whose purpose was to provide guidance and recommendations on injury prevention to the provincial advisory and executive committees. The Committee developed provincial injury prevention priorities using a mixed-methods approach to achieve group consensus and to reduce unintended results bias (Evans et al. 2018). These priorities were then approved by the Prevention and Health Promotion Policy Advisory Committee, BC Ministry of Health and included: 1) the prevention of falls and fall-related injuries among older adults ages 65+ years; 2) transport-related injury prevention, specifically among motor vehicle occupants, cyclists, pedestrians, and young drivers ages 16 to 24 years; and, 3) the prevention of self-harm among youth ages 15 to 24 years (BC Injury Prevention Committee (BCIPC) 2017, Evans et al. 2018). Overall injury hospital separation rates that included both unintentional and intentional injuries, as well as the hospital separation rates in each of the priorities were identified as priority indicators that could provide evidence of health disparities in BC. Using these indicators, the purpose of this study was to examine disparities across neighbourhood socioeconomic levels for injury hospital separations overall, as well as within each priority area, so as to better understand and develop more precise prevention initiatives, particularly for the more vulnerable populations in BC.

## Methods

Acute hospital separations for the period April 1, 2009 to March 31, 2014 were obtained from the Discharge Abstract Database (DAD), BC Ministry of Health. Hospital separations were identified for all external causes of injury, and for the three BC injury prevention priorities, using the International Statistical Classification of Diseases and Related Health Problems Canadian version 10 (ICD-10-CA) codes V01 to Y36 and Y85 to Y89 (Table 1). Hospital separations due to medical or surgical complications, adverse effects from the therapeutic use of medications, and after effects of injury were excluded. The DAD data do not include socio-economic information; therefore, an ecological approach was applied and socio-economic level of the injured individual’s area of residence was attributed to each injury hospital separation case.

**Table 1:** Classification of injuries and BC priorities using ICD-10-CA

Overall Injuries	V01-Y36; Y85-Y89
Transport-related	
Motor Vehicle Occupant	V30-V79; V87-V89
Pedestrian	V01
Cyclist	V10
Young drivers, 16–24 years	V30 – V79; .0, .4, .5
Falls, 65+ years	W00-W19
Youth Self-harm, 15–24 years	X60-X84; Y870

The most granular geographical unit available for this study using DAD data was the Forward Sortation Area (FSA), which is a geographic region based on the first three characters of the 6-digit Canadian postal code. Three variables from the 2011 CensusPlus data that best represented material deprivation were utilized in the analysis (Gagne and Hamel 2009, Pampalon et al. 2009). The economic and poverty variables (EPVs) included: average household income (income); proportion of individuals among the population aged 15 and over with at least a high school diploma (education); and proportion among the population aged 15 and over who were employed (employment). Age standardized rates per 100,000 FSA population were calculated using the direct method of standardization, where the average annual number of hospital separations over the five-year period was divided by the FSA population, as obtained from the 2011 CensusPlus. The 2011 Canadian population was used as the standard population to account for differences in the age distribution. Age specific rates per 100,000 FSA population were also calculated for specific age groups and causes using the average annual number of hospital separations over the five-year period, divided by the FSA population, as obtained from the 2011 CensusPlus. Cases of injury hospital separations with missing or unknown FSA, and sex were excluded for all analyses.

Rate ratios, with 95% confidence intervals (CI), were calculated for overall injury hospital separations and each of the BC injury prevention priorities by fitting seven independent Poisson regression models. Number of injury hospital separations was used as the outcome variable, with a log-population offset and the three EPVs as predictor variables, adjusted for sex, age and health region for overall injury hospital separations together with separate models for each of the BC injury prevention priorities. Holm-Bonferroni correction was used to correct for the seven models tested to maintain an alpha level of 0.05. All analyses were conducted using SPSS version 23.

## Results

Between April 1, 2009 and March 31, 2014, there was a total of 178,626 injury-related hospital separations, however 765 (0.4%) of these had FSA missing and unknown sex, and were therefore excluded from the analysis. Following exclusion, a total of 177,861 injury-related hospital separations between April 1, 2009 and March 31, 2014 remained, averaging 35,572 hospital separations per year, and an annual average rate of 779.0 injury hospital separations per 100,000 population (Table 2). The average number and hospital separation rate by sex, age and BC injury prevention priorities are presented in Table 2. Injury hospital separation data were available for 191 FSAs in BC. The EPV variables from CensusPlus were available for 189 of the FSAs. The population in these FSA’s ranged between 31 to 106,114 individuals. The distribution of the three EPVs varied by FSA, with a mean average household income of $83,100, mean proportion of individuals aged 15 and over with at least a high school diploma at 93%, and mean proportion of individuals aged 15 and over who were employed at 27% (Table 3).

**Table 2:** Average annual number and hospital separation rate by sex, age and BC injury prevention priorities

	Number^a^	Rate^b^	95% CI
Total overall injuries	35,572	779.0	(770.8-787.3)
Males	17,175	768.5	(756.9–780.2)
Females	18,398	797.7	(786.1–809.4)
Age Group			
0–14	1952	288.2	(275.5–301.0)
15–19	1574	572.0	(543.8–600.2)
20–24	1674	598.2	(569.7–626.8)
25–44	5865	505.9	(492.9–518.8)
45–64	8063	611.0	(597.7–624.3)
65–74	3935	1058.9	(1026.0–1091.8)
75–84	5914	2635.1	(2568.9–2701.4)
85+	6596	7184.6	(7017.5 – 7351.6)
BC Priorities			
Transport-related			
Motor Vehicle Occupants	1786	40.0	(38.1–41.8)
Pedestrians	556	12.4	(11.4–13.5)
Cyclists	876	20.0	(18.6–21.3)
Young drivers, 16–24 years	152	28.4	(23.9–32.9)
Falls, 65+ years	13,368	1943.5	(1910.9–1976.1)
Youth Self-harm, 15–24 years	683	123.0	(113.8–132.3)

^a^Average numbers are rounded. ^b^Note: Rate standardized per 100,000 population for all, except by age groups, using Canada 2011 Population

**Table 3:** Distribution of area level economic and poverty variables of interest by FSA

	Mean	Median	Standard deviation	Range	
Income	$83,100	$76,500	$27,800	$30,600	$251,900
Education (%)	92.76	93.02	2.89	82.80	99.40
Employment (%)	26.59	26.63	3.24	16.54	34.70

### Overall injury hospital separations

Across all causes of injury, hospital separation rates were found to be significantly associated with all three neighbourhood levels of EPVs: income, education and unemployment (Table 4). An inverse relationship was found between injury hospital separation rates and neighbourhood levels of income, where injury hospital separation rates decreased by 2.1% (95% CI: 1.8–2.4) for every $10,000 increase in FSA levels of average household income. An inverse relationship was also found for neighbourhood levels of education, where injury hospital separation rates decreased by 1.8% (95% CI: 1.6–2.1) for every 1% increase of at least a high school education in the FSA.

**Table 4:** Rate ratios for Poisson regression modelling for all injury hospital separations and BC priorities

	All Injuries		MV Occupants		Cyclists		Pedestrians		Falls 65+		Youth Self Harm		Young Drivers	
Variable	Rate Ratio (95% CI)	*p*-value	Rate Ratio (95% CI)	*p*-value	Rate Ratio (95% CI)	*p*-value	Rate Ratio (95% CI)	*p*-value	Rate Ratio (95% CI)	*p*-value	Rate Ratio (95% CI)	*p*-value	Rate Ratio (95% CI)	*p*-value
Sex^a^	**1.021 (1.011, 1.031)**	< 0.001	**1.257 (1.206, 1.311)**	< 0.001	**3.443 (3.208, 3.694)**	< 0.001	**1.204 (1.117, 1.297)**	< 0.001	**0.468 (0.460, 0.476)**	< 0.001	**0.433 (0.402, 0.465)**	< 0.001	**1.887 (1.625, 2.190)**	< 0.001
Region^b^														
Interior	**1.153 (1.129, 1.178)**	< 0.001	**1.308 (1.204, 1.421)**	< 0.001	**1.893 (1.614, 2.220)**	< 0.001	1.225 (1.004, 1.494)	0.045	**1.258 (1.208, 1.309)**	< 0.001	1.087 (0.941, 1.255)	0.257	**1.494 (1.117, 1.998)**	0.007
Fraser	1.007 (0.987, 1.027)	0.505	**0.874 (0.809, 0.944)**	0.001	1.091 (0.936, 1.272)	0.265	**2.054 (1.716, 2.457)**	< 0.001	**1.382 (1.329, 1.437)**	< 0.001	**0.843 (0.740, 0.961)**	0.011	0.824 (0.631, 1.075)	0.154
Vancouver Coastal	**0.836 (0.818, 0.855)**	< 0.001	**0.566 (0.516, 0.621)**	< 0.001	**1.605 (1.366, 1.887)**	< 0.001	**1.809 (1.493, 2.194)**	< 0.001	**1.126 (1.080, 1.175)**	< 0.001	**0.567 (0.485, 0.662)**	< 0.001	**0.323 (0.226, 0.462)**	< 0.001
Island	0.981 (0.959, 1.003)	0.091	**0.808 (0.734, 0.890)**	< 0.001	**1.739 (1.473, 2.052)**	< 0.001	1.227 (0.998, 1.508)	0.052	**1.123 (1.077, 1.172)**	< 0.001	1.006 (0.861, 1.174)	0.943	0.878 (0.625, 1.233)	0.451
Age^c^	**1.190 (1.189, 1.192)**	< 0.001	**1.085 (1.080, 1.090)**	< 0.001	**0.978 (0.972, 0.985)**	< 0.001	**1.078 (1.069, 1.087)**	< 0.001	**1.530 (1.521, 1.539)**	< 0.001	**0.803 (0.750, 0.859)**	< 0.001	**1.680 (1.450, 1.945)**	< 0.001
Income (per $10,000)	**0.979 (0.976, 0.982)**	< 0.001	**1.022 (1.008, 1.036)**	0.001	1.006 (0.991, 1.022)	0.426	**0.888 (0.867, 0.910)**	< 0.001	**0.976 (0.972, 0.980)**	< 0.001	0.988 (0.967, 1.009)	0.248	1.043 (0.993, 1.096)	0.095
Education (per % of population)	**0.982 (0.979, 0.984)**	< 0.001	**0.889 (0.878, 0.900)**	< 0.001	1.017 (1.000, 1.035)	0.054	**0.971 (0.952, 0.992)**	0.006	**1.040 (1.036, 1.045)**	< 0.001	**0.952 (0.933, 0.971)**	< 0.001	**0.873 (0.836, 0.912)**	< 0.001
Employment (per % of pop)	**1.006 (1.004, 1.008)**	< 0.001	**1.033 (1.023, 1.043)**	< 0.001	1.011 (0.998, 1.025)	0.088	1.011 (0.994, 1.028)	0.198	**0.995 (0.992, 0.998)**	0.004	0.996 (0.980, 1.012)	0.583	1.022 (0.985, 1.060)	0.251

Bold represents rate ratios that were significant after correction. ^a^compared to females ^b^compared to Northern ^c^based on five-year age increments

A direct relationship was found between injury hospital separation rates and neighbourhood levels of employment; hospital separation rates increased by 0.6% (95% CI: 0.4–0.8) for every 1% increase of employment in the FSA population.

### Falls among 65 years and older

Injury hospital separation rates due to falls among older adults were found to be significantly associated with neighbourhood levels of income, employment, and education (Table 4). Inverse relationships were found for neighbourhood levels of income and employment, where injury hospital separation rates decreased by 2.4% (95% CI: 2.0–2.8) for every $10,000 increase in the FSA level of household income; and decreased by 0.5% (95% CI: 0.2–0.8) for every 1% increase in employment in the FSA population.

A direct relationship was found for neighbourhood levels of education, where injury hospital separation rates increased by 4.0% (95% CI: 3.6–4.5) for every 1% increase of at least a high school education in the FSA population.

### Motor vehicle occupants

Injury hospital separation rates among motor vehicle occupants were found to be significantly associated with neighbourhood levels of income, employment and education (Table 4). Direct relationships were found for neighbourhood levels of income and employment, where injury hospital separation rates increased by 2.2% (95% CI: 0.8–3.6) for every $10,000 increase in FSA level of average household income; and increased by 3.3% (95% CI: 2.3–4.3) for every additional 1% of employment in the FSA population.

An inverse relationship was found for neighbourhood levels of education, where injury hospital separation rates decreased by 11.1% (95% CI: 10.0–12.2) for every 1% increase of at least a high school education in the FSA population.

### Cyclists

Injury hospital separation rates among cyclists were not found to be significantly associated with neighbourhood levels of income, employment, nor education (Table 4).

### Pedestrians

Injury hospital separation rates among pedestrians were found to be associated with neighbourhood levels of income and education, while there was no significant association with employment (Table 4). Inverse relationships were found for neighbourhood levels of income, where injury hospital separation rates decreased by 11.2% (95% CI: 9.0–13.3) for every $10,000 increase in FSA level of average household income; and for neighbourhood levels of education, where injury hospital separation rates decreased by 2.9% (95% CI: 0.8–4.8) for every 1% increase of at least a high school education in the FSA population.

### Young drivers

Injury hospital separation rates among young drivers were found to be significantly and inversely associated with neighbourhood levels of education, where injury hospital separation rates decreased by 12.7% (95% CI: 8.8–16.4) for every 1% increase of at least a high school education in the FSA population (Table 4). No significant associations were found for young drivers with neighbourhood levels of income or employment.

### Youth self harm

Injury hospital separation rates for youth self-harm were found to be significantly and inversely associated with neighbourhood levels of education, where injury hospital separation rates decreased by 4.8% (95% CI: 2.9–6.7) for every 1% increase of at least a high school education in the FSA population (Table 4). No significant associations were found for youth self-harm with neighbourhood levels of income or employment.

## Discussion

This ecological analysis clearly demonstrates that injury hospital separation rates vary with the measured neighbourhood area SES variables, namely income, education, and employment. In British Columbia, injury hospital separation rates in general demonstrated an inverse relationship with neighbourhood levels of income and education, where individuals from deprived areas were at a greater risk of injury hospital separations than individuals from privileged areas. Individuals living in deprived areas may have increased exposure to hazards due to higher density traffic, poor road conditions and infrastructure, poorer housing, more criminal activity, less access to fire and police protection, and fewer well-maintained recreational facilities, all of which can increase the risk of injury (Pickett and Pearl 2001, Kohen et al. 2002).

The results also suggest neighbourhood area SES differences were associated with the injury hospital separation rates for the three BC injury prevention priorities, namely, falls among older adults; motor vehicle crashes involving motor vehicle occupants, pedestrians, cyclists and young drivers; and, youth self-harm. Previous studies have shown a strong inverse association with increasing neighbourhood SES and injuries (Cubbin and Smith 2002, Lawson et al. 2015). A review of 70 census-based socioeconomic indicators for monitoring injury found that not all indicators of SES were specifically relevant to injury and that a strong association for neighbourhood area SES was observed in conjunction with level of education (Bell et al. 2015). The authors of this review found that income constructs were less consistent in showing associations with injury hospital separations than demographic and occupational constructs. This study found the FSA level of education was strongly associated with injury hospital separations in most of the models analysed, indicating that this is a good measure of neighbourhood area SES with injury risk.

Within BC’s public health system, *Promote, Protect, Prevent – BC’s Guiding Framework for Public Health and Healthy Families BC Policy Framework* (The Guiding Framework) identifies priorities where key public health challenges are addressed and strategic investments made to address these challenges. Goal 5 in the Guiding Framework speaks to making BC a safer province that reduces the risk of preventable injuries and includes the following objective:

*Build a culture of safety at work, home and play by increasing awareness of injury risks, implementing prevention education and taking priority actions, such as designing and developing safe environments, systems and products*. (BC Ministry of Health 2017).

Based on the recommendations made in The Guiding Framework (2017), the BCIPC was formed and injury priorities were identified. The analysis conducted for the BC injury prevention priorities showed that lower levels of education within FSAs was associated with lower fall-related injury hospital separation rates among older adults. This is a surprising finding, as an inverse relationship was expected. Few studies have examined this effect and this finding may be attributed to FSA’s with low levels of education being more densely populated and having a greater proportion of people living in households, allowing for more individuals to be available to care for the elderly. Conversely, those with higher education may remain more physically active and engage in exercise and recreation, and work independently, therefore exposing themselves to greater risks of falling while engaging in these activities.

*BC’s Road Safety Strategy: 2015 and Beyond* defines a collaborative process among all road safety partners with the goal to have the safest roads in North America, highlighting the importance of encouraging innovation and flexibility using a *Safe System Approach* (RoadsafetyBC 2016). More recently, the *BC Road Safety Strategy* has moved towards a *Vision Zero* movement to eliminate motor vehicle crash fatalities and serious injuries, achieved by targeting key areas of concern using the *Safe System Approach*. To address the road safety priorities identified for BC, the BCIPC is committed to work on initiatives that adhere to *Vision Zero*. The results of this study provide useful information to achieve *Vision Zero,* particularly pertaining to the built environments in poor neighbourhoods were motor vehicle occupants, pedestrians and cyclists may be injured as a result of unsafe speeding behaviours, higher density traffic, poor road conditions and infrastructure as well as lack of safe pedestrian walkways and crossing.

This analysis demonstrated that injury hospital separation rates among motor vehicle occupants increased with a neighbourhood area increase in income and employment. This may be attributed to individuals in high income and employment FSAs being more likely to be able to afford cars, particularly, young drivers who might access family resources or be gifted a car. It should be noted that the injury risks calculated were based on total population and not per vehicle or driver, therefore not depicting true rates. Previous studies have found an inverse relationship between education and traffic crashes where individuals in neighbourhoods with low education levels experienced higher rates of injuries in traffic crashes (Sami et al. 2013, van Lenthe et al. 2004). This finding is consistent with the results of our study, where motor vehicle occupant hospital separation rates decreased with a neighbourhood level increase in education. The combination of neighbourhood levels of low education, and low employment, and low income creates barriers for motor vehicle ownership. Individuals in neighbourhoods with higher income and employment levels may have greater access to vehicles, hence greater risk for motor vehicle occupant injuries. This is supported by findings from a study where decreasing level of neighbourhood education was associated with an increasing percentage of individuals who do not use a car (van Lenthe et al. 2004).

This study showed that FSAs with higher income and higher education had significantly lower injury hospital separation rates among pedestrians, similar to findings in Chakravarthy et al. (2010), that showed an increase in pedestrian crashes within poor, low income neighbourhoods. Impoverished FSAs may be closer to busy streets and industrial sites with limited safe play areas or parks, creating higher risks for crashes and injuries. Additionally, impoverished neighbourhoods may have lower vehicle ownership and a greater number of pedestrians, creating a higher exposure for pedestrian injuries.

The results from this study show that higher hospital separation rates among young drivers were associated with FSAs with lower levels of education. This is consistent with other studies that have found that young drivers in neighbourhoods with higher education were underrepresented in all crash patterns (Laflamme et al. 2006), and that the high school completion rate in the neighbourhood was significantly associated with a decreasing pattern of risky driving behaviour, including speeding, restraint use non-compliance, impaired driving, and driving when fatigued (Vassallo et al. 2016). Youth in neighborhoods with low level of education conducting such risky driving behaviours were more prone to crashes.

Finally, this study found that FSAs with lower levels of education were associated with higher self-harm related hospital separation rates among youth. Highlighting the association of poor education levels in neighbourhoods with higher risk of youth self-harm is important, to inform policies to include interventions in these neighbourhoods such as enrichment programs for young children to keep them in school longer, and programming for youth enabling them to return to school or to access retraining opportunities in order to seek employment, enhance their living standards and enrich their environment.

Studies have reported that poorer educational achievement was most prevalent among those with suicidal self-harm (Mars et al. 2014, Rahman et al. 2018). The association between depression and self-harm among youth and low levels of educational attainment have been identified in several studies (Bjelland et al. 2008, Moilanen et al. 2010, Reiss, 2013). Both depression and self-harm were found to be associated with deprivation (Rahman et al. 2018). There is evidence to suggest that there are many social and familial risk factors as well as adverse family circumstances associated with self-harm, which include abuse, neglect and poor attachment. Families in areas with lower SES may often face such challenges resulting in higher prevalence of self-harm among youth in these areas. It has been shown that implementing programs that improve the educational attainment among youth, results in higher rates of school completion and leads to healthy behaviours and high income earning potential (Lochner 2010, Oreoupoulos et al. 2017).

### Data limitations

There were no personal identifiers included in the available data, which would have enabled the exclusion of hospital readmissions and transfers and the ability to limit the effects of variations related to service use. However, the average number of readmissions and transfers for injury makes up just a small proportion (8.5%) of the total number of injury hospital separations for the period under study. Medical care practices may vary in rural areas as a result of lack of availability of specialized care and training, as well as distant telehealth options, where injuries may be reported to physicians rather than to hospitals, and only the very severe injuries hospitalized. To account for these differences, it would have been instructive to conduct the analysis on severe injuries only – those that would always be hospitalized. However, severe injuries for all ages could not be identified. While a severe injury indicator for children and youth has been developed and is utilized in BC, (Pike et al. 2017), work to develop an indicator for severe injuries among adults and older adults is needed.

For the three BC injury prevention priorities, true rates could not be computed as data were not available that reflected the true number in the exposed population. This is particularly relevant for the transport-related injury hospital separations, as compared to falls among older adults and youth self-harm. However, the population rates used should adequately reflect the true exposure.

Data for the three EPVs were not available at the individual level but rather at a FSA level and were attributed to the individual, therefore an ecological fallacy is possible where an overall association found for an aggregate area is applied to the individual (Young 2005). However, this is a common limitation among studies using this approach and previous evidence has shown that neighbourhood area SES effects do reflect well the individuals’ social economic condition (Townsend et al. 1987).

In addition, many factors need to be considered when interpreting differences in deprivation, such as, economic conditions, nature of jobs, types and availability of social supports, and ethnic composition and culture. These factors vary across geographic regions and could not be accounted for in the current analysis.

## Conclusions

Previous studies have used deprivation indices that combine the individual components into an area-based composite score. This study was the first to quantify socioeconomic disparities in injury hospital separations among British Columbians in support of program planning and policy development related to provincial injury prevention priorities. Three specific measures of neighbourhood area SES, namely income, education, and employment were used to define material deprivation and have been applied to identify the vulnerable areas and gaps pertaining to injury hospital separations for all injuries, and for injuries pertaining to the three BC injury prevention priorities. The study results show that various mechanisms of injury have separate and different relationships with neighbourhood levels of income, education and employment.

Consistent with previous research, the results of this study suggest that neighbourhood area SES is associated with the risk of injury hospital separations. In particular, low education levels in FSAs was associated with increased risk of injury hospital separations, mainly for motor vehicle occupants, pedestrians, young drivers, and youth self-harm. The results of this study provide useful information for implementing injury prevention initiatives and interventions in BC to address the identified injury prevention priorities, and which align with both the goals of The Guiding Framework and RoadSafetyBC’s *Vision Zero*. The results provide meaningful information to the BCIPC as they embark on the next steps of injury prevention in BC.

## Abbreviations

BC: British Columbia
BCIPC: BC Injury Prevention Committee
CI: Confidence intervals
DAD: Discharge Abstract Database
EPV: Economic and poverty variables
FSA: Forward Sortation Area
ICD-10 CA: International Classification of Diseases and Related Health Problems
SES: Socio economic status
